# Macrophages at the Fork in the Road to Health or Disease

**DOI:** 10.3389/fimmu.2015.00059

**Published:** 2015-02-16

**Authors:** Charles D. Mills, Laurel L. Lenz, Klaus Ley

**Affiliations:** ^1^BioMedical Consultants, Marine on St. Croix, MN, USA; ^2^Department of Immunology and Microbiology, University of Colorado School of Medicine, Aurora, CO, USA; ^3^La Jolla Institute for Allergy and Immunology, La Jolla, CA, USA

**Keywords:** macrophage, innate immunity, M1, M2, wound, cancer, infection, atherosclerosis

Macrophages are the epicenter of all immune systems (1). The first and the most abundant leukocyte observed (2), macrophage have long been relegated to the role of “servants” of T or B cells/adaptive immunity. This view is now known to be backward. Macrophages necessarily initiate and direct virtually all immune responses from simple multicellular animals to humans.

There is good news and bad news in the newly recognized importance of macrophages/innate immunity. The well-known “double-edged sword” nature of the immune system can largely be attributed to macrophages’ unique ability to make polar-opposite repair/heal (M2) or kill/inhibit (M1) type responses (3).

In health, M2-type macrophages maintain homeostasis by helping repair and replace lost or effete cells. Ever-present in tissues, macrophages are also the primary host defense against pathogens (or altered self cells) because their unique physiology allows them to rapidly switch from their M2/heal mode to an M1/inhibit mode: both powerful responses; both potentially dangerous.

In disease, over expression of M2/heal macrophages contributes to chronic infections, fibrosis, allergy, and cancer (3). Conversely, M1/inhibit-dominant activity plays a major role in atherosclerosis, autoimmunity, and other chronic inflammatory conditions.

Of fundamental importance is that both the routine M2/heal and the induced M1/inhibit macrophage functions occur in all animals whether they have T cells or not. Furthermore, M1 and M2 macrophage responses play necessary roles in causing T cells to make Th1 or Th2-type responses if pathogens or altered self are present (4). Hence, the renaming of macrophage responses M1 and M2.

This new knowledge about the central role of macrophages in immune systems brings great promise for increasing health/decreasing disease. In this regard, the ability of macrophages to exhibit the polar-opposite M2/heal and M1/kill functions result, in part, from their unique ability to metabolize one amino acid – arginine – to either growth-promoting ornithine or growth-inhibiting nitric oxide (NO) (5). Hence, the title of this Topic, “M1 and M2 Macrophages: The Arginine Fork in the Road to Health and Disease.” We hope that the articles assembled here help illuminate the basic functions of macrophages referred to as SHIP [sample, heal, inhibit, and present (antigen)]. Such knowledge is critical for developing the means to modulate the direct M2/heal or M1/inhibit responses of macrophages, or their indirect abilities to initiate and direct T and B cell responses. One can properly say macrophages are the “chicken and the egg” of immunity (1).

## Origin of M1/Inhibit and M2/Heal Macrophages and the Scope of the Topic

As an introduction to M1 and M2 macrophages, a chronology of results (and publications) that led to their discovery is summarized below:

- Macrophages have the unique ability to produce a growth-inhibiting molecule (NO) or a growth-promoting molecule (ornithine), through the enzymatic conversion of l-arginine in different ways (6–8).
- Macrophages in normal tissues, healing wounds, or in growing tumors metabolize arginine primarily to ornithine via arginase (later to be named M2-type). Macrophages can switch to producing NO via iNOS (to be named M1-type) that is necessary to kill cancer or many pathogens. Arginine is the source of both ornithine and NO (9–12).
- Macrophages were specifically renamed M1 and M2 to highlight that innate immunity controls adaptive immunity. M1 (NO) or M2 (ornithine)-type macrophage responses are T cell independent and they stimulate Th1-type and CTL responses, or Th2-type responses, respectively (1, 3–5). Thus, M1/M2 represents a sea change in our understanding of how immune responses occur.

These studies have stimulated thousands of publications that have enhanced our knowledge of the importance of M1/inhibit and M2/heal functions, and other cytokines and factors that accompany these responses (1). Here, we have assembled papers by contributors that focus on basic aspects of macrophage biology, their roles in various diseases, and how they are regulated. Macrophages evolved long before other immune cell types and are the foundation of all animal immunity (13). Therefore, we begin with a series of “introductory” articles where readers can find basic information about macrophage biology and functions. These articles also trace the evolutionary origins of macrophages to aid in understanding their central role in immune systems. Next, articles detail the roles of macrophages in protection against (or causation of) various diseases including wounds, cancer, infections, atherosclerosis, obesity, hypertension, and other conditions. Finally, we look to the future with several articles detailing how macrophage M1/inhibit and M2/heal functions might be modulated for therapeutic benefits. We hope that the articles enhance your knowledge of this singularly multitalented and remarkable leukocyte.

## Introduction to Macrophage Biology and Functions

To better appreciate macrophages, it is useful to known about their unique biochemistry, functions, and central role in all immune systems. Drs. Rath and Munder provide a comprehensive biochemical introduction to macrophage arginine metabolism, and how these cells can take “the fork in the road” to make either an arginine-based M1/inhibit or M2/heal response (14). Because “Nothing makes sense except in the light of evolution” (Theodosius Dobzhansky), Dr. Buchmann traces the evolutionary origins of both innate and adaptive immunity, and shows when new macrophage (and other) immune functions evolved, culminating in humans (15). Dr. Dzik importantly reveals that the macrophage M2/heal function (arginases) preceded the M1/inhibit function in animal evolution (16). Drs. Mills, Thomas, Lenz, and Munder describe the basic “SHIP” functions of macrophages [sample, heal, inhibit, and present (antigen)], and why it is important to study these functions to understand macrophage biology *in vivo* (17). In a similar vein, Drs. Italiani and Boraschi elucidate why examining macrophages by functions versus “phenotypes” can be critical for understanding how they affect health (18). Dr. Harris’ piece colorfully describes the two-edged sword nature of macrophages as “The Good the Bad and the Ugly” phases of inflammation. He also illuminates that M1/inhibit or M2/heal-type macrophage activities vary enormously in different microenvironments of lungs or other sites of inflammation (19). In turn, important local functions can be lost if one grinds up whole organs as is common. Drs. Thomas and Mattila provide an in-depth look at macrophage arginine metabolism in different vertebrate species (20). Importantly, they show that, contrary to some reports (21, 22), macrophages in mice and humans are quite similar, as one might expect from analyzing evolution (e.g., Drs. Buchmann and Dzik, mentioned earlier).

## Macrophage Influences in Different Diseases

### Wounds and cancer: stark examples of the two-edged sword nature of macrophage responses

Wound healing requires M2/heal-type responses (9). If M1/inhibit responses occur (e.g., infected wounds) wound healing is delayed until the infection is cleared (5). M2/heal-type macrophages also dominate inside tumors in experimental animals and humans (5, 11, 23). In marked contrast to their beneficial effect in wounds, M2-type macrophages actively promote tumor growth [reviewed in Ref. (3)], in part, by secreting growth factors (11, 24, 25).

Regarding the relative roles of M1 or M2-type macrophages in wounds or implanted biomaterials, Drs. Brown, Sicari, and Badylak demonstrate that there is 2–3 day dominance of M1-type macrophages (26). These data support that the first biologic priority of hosts following injuries is to prevent infections (5). However, if an injury is sterile, the priority switches to M2-type dominant macrophages that are necessary for proper healing (17). Interestingly, they note that biomaterials with larger pore sizes have less scarring/fibrosis. Thus, the physical properties of implanted materials seem important in allowing functional regeneration over typical imperfect wound healing found in adult humans (27). Drs. Beljaars, Schippers, Smit, Martinez, Helming, Poelstra, and Melgert compared M1- and M2-types of macrophages inside chemically damaged livers in mice and cirrhotic livers in humans (28). The liver is one of the few organs able to regenerate (though not perfectly) (29). So, it was interesting that they found a predominance of M1 macrophages during resolution of damage, which contrasts with M2 macrophages that dominate in wounds elsewhere, and which results in scarring/healing, as described. Also, interesting was that the authors observed distinct M1 and M2-type macrophages in close proximity to each other with little overlap in markers. These findings do not support the hypothesis that macrophages only resemble a “color wheel” with multiple overlapping characteristics (30).

In contrast to the beneficial effects of M2-type macrophages in wounds, these same types of macrophages promote cancer growth and metastases as mentioned [reviewed in Ref. (3)]. Why is there cancer, and why does the immune system help it grow?

Species successfully evolve by acquiring traits that provide survival advantages, and losing undesirable traits. Environmental and societal influences aside, the way animals (any species) change heritable traits is through producing progeny: breeding. Cancer in humans occurs mainly after breeding age. So, there has been little evolutionary pressure (or advantage) for humans to acquire traits that prevent cancer, or that could stop it if it appears. The same lack of evolutionary pressure applies to atherosclerosis, or many other “adult” diseases that mainly occur during post-breeding (to be discussed later). Too, mate selection (important in breeding success) is mostly unaffected by knowledge of whether parents or grandparents died of cancer or other late-appearing diseases (3).

Once it appears, cancer can be eliminated. How? Modulating macrophage functions. It is now known that the majority leukocytes in tumors are macrophages: sometimes >50% of a tumor mass. However, as mentioned, these tumor-associated macrophages (TAM) are primarily M2-type and actively promote tumor growth: Cancer is often referred to as “a wound that doesn’t heal” (31). But, a wealth of recent evidence indicates that decreasing M2/heal-type and increasing M1/inhibit-type macrophages can slow or reverse tumor growth (11, 32). This is an exciting development because conventional immunologic thinking purports that tumors need to be recognized as “foreign,” like a pathogen. But, most tumors are not “foreign.” So, it falls on the shoulders of innate immunity, not adaptive immunity (T and B cells), to stop cancer. Importantly, recent antitumor effects being observed seem primarily (or solely) mediated by macrophages/innate immunity, not T cells (32). Even if a human cancer is recognized as “foreign,” it is still critical to switch M2- to M1-like macrophages. This is so because of the new knowledge, discussed earlier, that M1-type macrophages are necessary to stimulate T cells to make tumoricidal Th1-type cellular killer responses such as CTL and further amplify M1/inhibit macrophages (1, 4). In a related connection, significant prolongation of survival in human cancer has recently been observed by inhibiting immunoregulatory molecules, such as PD-1 and CTLA4 (33). The effects observed have been postulated to involve specific anti tumor T cell activity. Such effects likely depend on modulating macrophage responses. Thus, increasing our knowledge of M1 and M2 polarization in cancer, and how to modulate it, is very important.

Drs. Laoui, Van Overmeire, Baetselier, Van Ginderachter, and Raes review the evidence that M2-type macrophages predominate in most human tumors with the notable exception of colorectal cancer (34). They also describe new evidence that colony-stimulating factors are important players in determining the quantity and type of macrophages that populate tumors. MCSF is normally present in tissues and plasma, and is associated with M2-type macrophages. In contrast, GM-CSF is only present following injury or during infections, and is associated with M1-type responses. Interestingly, the authors highlight findings suggesting inhibition of MCSF by various means in humans does not only simply decrease M2-type TAM but also increases the M1/M2 ratio. Thus, altering macrophage differentiation signals can affect macrophage polarization beneficially in clinical settings. Using a different approach, Drs. Fritz, Tennis, Orlicky, Lin, Ju, Redente, Choo, Staab, Bouchard, Merrick, Malkinson, and Dwyer-Nield show that treatment of lung cancer in mice with a macrophage-depleting agent (clodronate-encapsulated liposomes) significantly decreases tumor burden (35). They also show that this treatment stimulates lung TAM that have a mixed M1- and M2-type phenotype suggesting that this depletion modality (like MCSF inhibition) may also increase the M1/M2 TAM balance. Together, these studies, like many others, are indicating that there are real and important clinical benefits from immunologically manipulating macrophage functions in cancer.

### Macrophages in infections

In the context of animal models of bacterial infection, M1-type macrophages and NO production are often, but not exclusively (36), associated with host protection (17). Conversely, M2-type macrophages are typically associated with bacterial persistence. An article by Drs. Ka, Daumas, Textoris, and Mege reviews macrophage polarization in infectious diseases (37). They discuss some difficulties encountered when trying to extend these concepts to bacterial infections in humans. In humans suffering from leprosy or Whipple’s, macrophage, M2-type polarization can be readily observed. However, as mentioned earlier, analyzing whole organs can overlook microenvironmental differences in inflammation (19). Also, studies with human patients often utilize peripheral blood monocytes that lack the polarized M1- or M2-type functions associated with tissue macrophages. They also review that many pathogens, such as *Leishmania*, can survive or spread by blocking or subverting the process of macrophage development toward an M1/inhibit phenotype (27). An article from Drs. Burrack and Morrison discusses how macrophage activation and arginine metabolism by M1- or M2-type macrophages can have diverse effects on health and disease during viral infections (38). Macrophage NO production during viral infections, as in other settings, can be induced independent of lymphocytes (4). The production of NO can have direct anti-microbial effects on certain bacteria, fungi, and viruses. Hence, the M1/inhibit phenotype in these settings plays an immune protective role. However, because NO kills non-specifically, it can also have immunopathologic or immune suppressive effects during infections by influenza, herpes simplex virus-1, and cytomegalovirus. Similarly, the two-edged sword nature of M2/heal macrophage responses can cut both ways. For example, M2/heal responses (via arginase and growth-promoting ornithine) usefully promote tissue repair in some viral infection models, and via stimulating protective antibody responses. However, M2/heal responses are also associated with viral persistence or immune pathology during many infections, such as coronavirus-induced sudden acute respiratory syndrome (SARS), hepatitis B or C viruses, Ross river virus, HIV, and influenza. These articles indicate that it is important to understand the infectious disease type, stage, and severity in order to properly modulate M1- or M2-type responses to optimally eliminate pathogens and decrease untoward pathology.

### Macrophage responses in atherosclerosis and other non-pathogen-induced inflammatory conditions

In contrast to the primarily protective role of M1/inhibit-type responses against infection agents, described above, these killer/damaging activities are often associated with bad outcomes in chronic inflammatory conditions.

Regarding atherosclerosis, Drs. Thomas and Mattila show that both M1- and M2-types of macrophages are found during foam cell formation, a hallmark of atherosclerosis (20). Drs. Hayes, Tsaousi, Gregoli, Jenkinson, Bond, Johnson, Bevan, Thomas, and Newby show that there is an altered expression of certain matrix metalloproteinases in atherosclerosis (39). Interestingly, they also show that M1 and M2 macrophage polarization in atherosclerotic ApoE null mice occurs in the absence of T- and B-lymphocytes, again highlighting the independence of innate immunity from adaptive immunity discussed earlier. Drs. Murphy, Dragoljevic, and Tall review the recent evidence that cholesterol efflux pathways regulate myelopoiesis (40). Traditionally, cholesterol efflux was considered as a safeguard against foam cell formation, but Tall’s group has shown that knocking out cholesterol efflux molecules like ABCA1 and ABCG1 cause profound changes in hematopoiesis associated with more Ly6C+ inflammatory monocytes and more neutrophils. This shift could lead to altered macrophage function. Drs. Peled and Fisher review dynamic aspects of macrophage polarization during atherosclerosis progression and regression: progression is associated with macrophage M1 polarization and regression with M2 polarization (41). The article by Drs. Yang and Ming looks at the less commonly studied arginase II enzyme. They show that, unlike arginase I, that is typically inversely related to macrophage NO production, arginase II seems under different regulation. Also, Arg II expression in endothelial cells tends to uncouple eNOS (or NOS1) causing loss of vascular tone (42).

Regarding other non-pathogen associated inflammatory conditions, Drs. Vlahos and Bozinovski review the role of alveolar macrophages in chronic obstructive pulmonary disease (COPD) (43). COPD is a widespread chronic inflammatory condition with immense medical and societal impact. Interestingly, in COPD, there is an accumulation of airway macrophages that show a transcriptome skewed toward wound healing M2 markers suggesting defective resolution of inflammation (as occurs in wound healing). Drs. Kraakman, Murphy, Jandeleit-Dahm, and Kammoun review macrophage polarization in obesity and type 2 diabetes (44). M1 “pro-inflammatory” macrophages are enhanced compared with M2 “anti-inflammatory” macrophages, leading to chronic inflammation and the propagation of metabolic dysfunctions. The brain and spinal cord are primarily populated by macrophage-like microglial cells, which are derived from yolk sac precursors under resting conditions. Drs. Cherry, Olschowka, and O’Banion show that these microglia are normally M2-polarized, although the microglia transcriptome is different from that of M2 macrophages in other organs (45). This observation fits well with an emerging concept that M1 and M2 polarization varies between organs (46). In this connection, Drs. Brown, von Chamier, Allam, and Reyes report on M1/M2 macrophage polarity in normal and complicated pregnancies and find that the balance and location of M1- and M2-type responses show significant variation (47). In general, over expression of M1-type macrophages is associated with untoward outcomes during pregnancy.

Finally, this Topic focuses on macrophage polarization. Little is currently known about how the origin of macrophages in tissues (e.g., yolk sac, Ly6C-high, Ly-6C-low monocytes) influence M1/M2 polarization. Drs. Dey, Allen, and Hankey-Giblin begin to explore how the ontogeny of monocytes and macrophage can influence M1- and M2-type responses in different tissues (48).

## Regulation of Macrophage Differentiation and Functions

The importance of macrophage M1/inhibit and M2/heal imbalances in various disease or protective processes being clear, immunologists and clinicians are interested in how they might therapeutically intervene to shape macrophage differentiation or modulate various macrophage functions to restore health. Drs. Wang, Liang, and Zen provide an overview of the various molecular mechanisms known to impact macrophage M1 and M2 polarization (49). Several of these mechanisms are expanded upon in subsequent articles. Cytokines such as interferon gamma (IFN-γ) have profound impacts on development of M1 and M2 functionality in macrophages. Regulators of cytokine receptor signaling can importantly impact macrophage functions. Dr. Wilson details in her article evidence that various suppressor of cytokine signaling (SOCS) protein family members shape M1/M2 macrophage functions in several disease settings (50). Colony-stimulating factors (CSFs) and macrophage stimulating protein (MSP) also impact macrophage responsiveness to polarization. Drs. Hamilton, Zhao, Pavicic, and Datta introduce the concept that the myeloid colony-stimulating factors MCSF and GM-CSF act not only to promote the development and maintenance of various myeloid populations but can also shape the responsiveness of macrophages to stimuli that direct the M1 or M2 phenotype (51). The effects of MSP and its receptor, a tyrosine kinase known as RON, on macrophage polarization are clearly presented in an article by Dr. Chaudhuri (52). Other extrinsic factors that regulate macrophage polarization include components of the complement cascade as well as extracellular nucleotides. The contrasting effects of various complement components on M1 and M2 functions are described in an article by Drs. Bohlson O’Conner, Hulsebus, Ho, and Fraser (53). Drs. Desai and Leitinger go on to detail how purinergic receptors for extracellular ATP and other nucleotides couple with calcium signaling to modulate macrophage activities and resolution of inflammation (54). An improved understanding of how these extrinsic factors and intrinsic signaling pathways regulate the acquisition of M1 and M2-type functions will lead to improved methods for fine-tuning of macrophage polarization to promote health.

As mentioned above, a key difference between M1 and M2 macrophages is in their processing of l-arginine. NO can not only kill susceptible microbes but also has a variety of signaling and regulatory effects on macrophages and other cell types. NO is generated from l-arginine through the activities of three NO synthase (NOS) enzymes. In contrast to canonical views, Drs. Mattila and Thomas present the perspective that “constitutive” enzyme activities (NOS1 and 3) can be induced, and “inducible” NOS2 is constitutively expressed in several tissues (55). The different functions of M1 and M2 macrophages are also associated with changes in the metabolic pathways they use to produce ATP. Drs. Galván-Peña and O’Neill discuss the differences in metabolism between M1 and M2 macrophages and how these differences impact other aspects of macrophage function (56). M1 macrophages mainly rely on glycolysis for energy, while M2 macrophages primarily use oxidative phosphorylation. Accumulation of succinate in M1 macrophages can stimulate HIF1α to sustain production of factors such as IL-1β and thus can impact the ability of M1 macrophages to prolong inflammation. Manipulation of NOS enzyme expression and activities as well as the products and consequences of the different metabolic processes in M1 and M2 macrophages should further our ability to shape the outcome of infections and other diseases.

## Summary

The “Fork in the Road” that macrophages take in making either M1/inhibit or M2/heal-type responses define “immunity” throughout the animal kingdom. In all animals, M1/inhibit-type responses are the primary host defense, and M2/heal-type responses help repair and replace lost or effete tissue to maintain host homeostasis. In humans (and other higher animals), macrophage M1/inhibit or M2/heal-type responses necessarily direct T (and B) cells/adaptive immunity to make Th1 or Th2-like responses. Thus, whether acting directly or indirectly, which “fork” macrophages take is the central controlling element that promotes health (as in pathogen control or wound repair) or impedes health (as in atherosclerosis, autoimmunity, or cancer). By illuminating the biochemical underpinnings, evolution, diseases, and regulation of macrophage functions, the papers in this Topic advance our understanding of how to modulate this most important of all leukocytes: the chicken and the egg of immunity (1).

## Conflict of Interest Statement

The authors declare that the research was conducted in the absence of any commercial or financial relationships that could be construed as a potential conflict of interest.
